# Adult, intensively socialized wolves show features of attachment behaviour to their handler

**DOI:** 10.1038/s41598-020-74325-0

**Published:** 2020-10-14

**Authors:** Rita Lenkei, Dóra Újváry, Viktória Bakos, Tamás Faragó

**Affiliations:** Department of Ethology, Eötvös Loránd University, Budapest, Hungary

**Keywords:** Animal behaviour, Social evolution

## Abstract

Dogs’ attachment towards humans might be the core of their social skillset, yet the origins of their ability to build such a bond are still unclear. Here we show that adult, hand-reared wolves, similarly to dogs, form individualized relationship with their handler. During separation from their handler, wolves, much like family dogs, showed signs of higher-level stress and contact seeking behaviour, compared to when an unfamiliar person left them. They also used their handler as a secure base, suggesting that the ability to form interspecific social bonds could have been present already in the common ancestor of dogs and wolves. We propose that their capacity to form at least some features of attachment with humans may stem from the ability to form social bond with pack members. This might have been then re-directed to humans during early domestication, providing the basis for the evolution of other socio-cognitive abilities in dogs.

## Introduction

Although the term attachment is often restricted to describe the relationship between parents and their infants^1–3^, the social bonds between romantic pairs or between the members of a social group like the family can be construed as forms of adult attachment where rather the perceived availability of the other individual matters^4,5^. In any case, the common ground is, that attachment is an individualized, affiliative relationship based on some degree of emotional dependency between two individuals enduring over time^6^. On the behavioural level, the attachment system manifests itself by preferences, what can be labelled as ‘functional attachment’ by fulfilling the following criteria: (a) individual recognition and preference for one individual (the attachment figure) over another; (b) exploration of the environment while the attachment figure is present (secure base); (c) stress reaction upon involuntary separation and seeking to re-establish the contact (separation stress and contact seeking); (d) seeking protection at the figure of attachment in danger (safe haven) and (e) intensive and specific greeting behaviour towards the figure of attachment after reunion^1,2,7^.

It was proven that dogs’ (*Canis familiaris*) behaviour towards their owner fulfils the above functional criteria of attachment^8,9^. Although, it is considered to be a crucial component of dog domestication, little is known about its evolutional origins. Though it is widely accepted that domestication and later the artificial selection caused genetic changes in the social competence of the dog^10,11^, it is unlikely that the capacity to form attachment bonds with humans has emerged as an entirely new trait, as changes during domestication are quantitative, rather than qualitative^12^. On the contrary, if attachment is based solely on socialization, then grey wolf (*Canis lupus*) pups—as the closest living relative—should show it similarly to dogs. Although research suggests that in some cases hand-reared and extensively socialized wolf pups show preference towards their caregiver^13^, the direct comparison of attachment behaviour of wolf and dog revealed that attachment behaviour is characteristic to dog puppies only at the age of 16 weeks^14^.

There is also little evidence about the filial origin of the dog–human relationship. Considering the social structure of canines and that the offspring are born immature, selective attachment towards the mother alone—at least at a very young age—would have not come with adaptive benefits for wolves^1^. First, attachment is less likely to develop in species in which the offspring do not leave the nest site, thus there is no need for the development of an individualised relationship. While in case of wolves, later they are left behind in the nest or den for longer periods since it would not be adaptive to show stress in the absence of their mother^15^. Second, the members of a wolf pack are usually closely related individuals, so that kin selection takes place. Consequently, alloparenting is common, which also works against the development of a specific mother–infant relationship and attachment^1,16^. Moreover, experimental studies failed to provide univocal explanation for their ability to form attachment towards their human caregivers during puppyhood^14,17^. Regarding the intraspecific filial attachment in dogs it was found that the mother and an unfamiliar female dog had the same effect as inferred from the reduction of stress induced vocalisations in separation^18^. Because of these contradictory results and that it seems that wolf and neither dog puppies form attachment towards their mothers, if the attachment towards the owner is formed yet it is unlikely that it stems from filial attachment towards the mother.

Even if it is broadly accepted that high sociability in the common ancestor of wolves and dogs had a major role in the evolution of the dog^19^, surprisingly, there is no theory how adult social behaviour of the ancient wolf might have influenced early domestication. While the size and the composition of a pack depends on several factors^20^, adult wolves typically live in families, in which the mating pair and their current pups are accompanied by offspring from earlier breeding seasons. These young stay with the pack for 1–3 years until they disperse to form new packs or to join another one^21^. Consequently, wolves live in extremely complex social environments in which mating pairs, leader–follower relationships and allegiances are formed, thus the emergence of strong individualised social bonds between the pack members is ecologically reasonable^27^.

In highly social mammals besides attachment there are other concepts explaining the comforting effect of other individuals in a stressful situation. Its most general form is the so called social buffering, when even an unfamiliar conspecific facilitates a faster recovery during aversive situations. This stress buffering effect is more pronounced when it is induced by a familiar individual compared to an unfamiliar conspecific^22^. While these phenomena are related with attachment and the secure base role of the attachment figure might be considered as a type of social buffering, attachment is a more complex phenomenon and it is distinguished from social buffering or from social familiarity. While in case of social familiarity the previous interactions with the conspecific also important in case of attachment what is emphasized is that individualised manner of the bond and the attachment figure is not interchangeable with any other familiar individuals^23,24^. For instance it was found that dogs distinguished between their owner and another familiar person in stress provoking situations^25^. The other main difference is that in case of attachment when the individual is involuntary separated from the attachment figure, it show separation stress, but it does not appears in case of social familiarity^23^. In dogs separation stress is well described and a highly investigated topic as it can be so intense that it represents a behavioural problem^26^.

While attachment was not experimentally tested between adult wolves yet there are some investigations suggesting its presence. It was found that captive wolves, when involuntarily separated from their pack, show behavioural and physiological signs of stress and contact seeking behaviour^27^. In line with this, if a member is separated from the pack, the other individuals have increased cortisol levels as a stress response. Moreover, the intensity of howling in pack members was in correlation with the strength of their affiliative relationship with the separated wolf^28^. It was also found that the presence of a sibling enhances the approach and explorative behaviour of a novel object more, than another pack mate’s suggesting that the relaxing effect of a familiar conspecific in a potentially stressful situation is also affected by the quality of the relationship^29^.

It is known that as a result of hand-rearing and intensive early socialization, wolf pups become attracted towards humans which is sustained into adulthood^13,30^. Ujfalussy and colleagues found that in a greeting situation, socialized wolves show more intense and friendly behaviours towards their original caregivers than to other familiar or unfamiliar people^30^. Although they did not discuss their results in the framework of attachment, individual recognition and intensive greeting behaviour are among its key features.

The widely used Strange Situation Test (SST), originally developed to observe human filial attachment^31^ was adapted to test dog-owner relationship^8^. However, the SST is not safely applicable in case of adult wolves as it involves a scenario when a stranger has to stay with the wolf alone in a confined space. As one of the SST’s main feature is the involuntary separation from the presumed attachment figure, separation tests are also widely used to study attachment (e.g. Refs.^32–34^). We developed an outdoor separation test similar to the SST in several aspects, i.e. an unfamiliar place and the presence of a stranger causing a moderate stress response that might activate the attachment behaviour. We tested whether adult, socialized wolves show features of attachment, including separation stress, contact seeking and secure base towards their handler. To have a reference for their attachment, we compared the wolves’ behaviour to that of normally socialized family dogs.

We tested N = 11 grey wolves and N = 9 family dogs. The wolves were more than 1.5-year-old, and individually hand-reared except for two that were also hand-reared but together with their siblings. One wolf due to deviations from the protocol and one individual’s second trial due to external disturbance had to be excluded, resulting in 10 wolves included in the analysis. The wolf tests were performed in an unfamiliar forest area while the dogs were tested in a silent area of the parking lot, next to the university buildings. Before the separation the Handler (H) and the Unfamiliar person (U) stood beside each other motionless, while one of them holding the leash, depending on the condition. After 30 s, the person who was assigned to leave, said “goodbye” to attract the attention of the wolf and walked away (Leaving phase). After a 50 m long walk along a path coming around a curve she disappeared from the view behind the vegetation. Following a 3-min period (Separation phase) she came back, greeted and petted the wolf (Return phase). During the test the person who stayed (U or H) with the wolf stood still—apart from marking the disappearance and reappearance of the leaving person with coughs—silently, avoiding any interaction. In the dogs’ tests the Owner played the role of the Handler, and the leaving person disappeared behind the building. Apart from the different locations, we kept the dogs’ and wolves’ test situations as similar as possible.

To compare the behaviour of the wolves and dogs and their reaction to the disappearing person, first using video-based behaviour coding (see Supplementary Table S3) we calculated scores by summing up the time percentage values of selected behaviours within Leaving and Separation phases separately, then averaged them over the two phases. The scores were (a) stress, containing whining, panting, other vocalizations and movement; (b) contact seeking, containing orientation at, and leash tension towards the Disappearing person; (c) exploration, containing sniffing the air and object exploration, (d) escape scale, containing general leash tension and chewing, (e) interaction, containing the sum frequencies of orientation at and physical contact with the Staying person. Finally, we analysed frequencies of mouth licking and pulling the leash separately.

## Results

**Figure 1:**
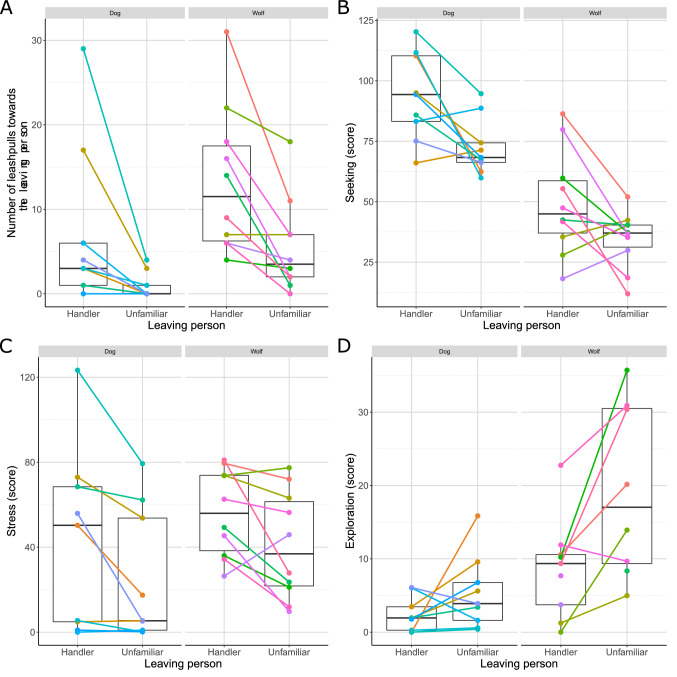
The behaviour differences between conditions when the Handler or the Unfamiliar person left the wolves and dogs. There were no interactions between the species and the leaving person, we used combined plots only for illustration sake. Scores can be higher than 100 due to the summing of different associated behaviours within phases. Boxplots show the medians, interquartile ranges and data range, while dots connected with lines show how the individual behaviours changed. Individuals are represented by different colours.

We found that when the H was leaving and eventually hid, both wolves and dogs were pulling the leash towards her more frequently (GzLMM of leash pulling frequency, leaving person:species interaction: LRT: χ^2^(1) = 10.443; p = 0.001; tukey post-hoc test, H → U in dogs: β ± SE = − 2.079 ± 0.368; z(17) = − 5.644; p < 0.001; in wolves: β ± SE = − 0.883 ± 0.159; z(17) = − 5.569; p < 0.001, Fig. 1A) and longer while also oriented longer in the direction of the hiding place (LMM of seeking: LRT: χ^2^(1) = 11.331; p < 0.001; H → U β ± SE = − 17.822 ± 4.797; t(18) = − 3.716; p = 0.002; Fig. 1B) what can be considered as contact seeking. While dogs showed more contact seeking behaviour than wolves (LMM of seeking: LRT: χ^2^(1) = 26.254; p < 0.001; D → W: β ± SE = − 41.089 ± 5.688; t(17) = − 7.224; p < 0.001), they pulled the leash less frequently towards the leaving person in general (leaving person:species interaction, tukey post-hoc test, D → W in H trial: β ± SE = 1.03 ± 0.456; z(17) = 2.260; p = 0.023; D → W in U trial: β ± SE = 2.23 ± 0.569; z(17) = 3.913; p < 0.001). Both species showed signs of separation stress as they moved, panted and whined significantly more (LMM of stress: LRT: χ^2^(1) = 14.170; p < 0.001; H → U β ± SE = − 17.512 ± 4.095; t(16) = − 4.276; p < 0.001; Fig. 1C) and also licked their lips more frequently (GzLMM of lip licking frequency LRT: χ^2^(1) = 13.868; p < 0.001; H → U β ± SE = − 0.644 ± 0.174; z(17) = − 3.691; p < 0.001) when the H left. Wolves somewhat habituated to the situation as they showed less stress signs in the second trial (LMM of stress: order: species interaction: LRT: χ^2^(1) = 4.835; p = 0.028; tukey post-hoc test, 1st → 2nd in dogs: β ± SE = 4.45 ± 5.89; t(16) = 0.756; p = 0.461; in wolves: β ± SE = − 13.260 ± 5.630; t(16) = − 2.356; p = 0.032) while they tended to lick their lip slightly more than dogs in both trials (GzLMM of lip licking frequency LRT: χ^2^(1) = 3.548; p = 0.060 trend only; D → W β ± SE = 1.085 ± 0.577; z(17) = 1.880; p = 0.06). In contrast, both wolves and dogs explored their vicinity significantly more if the H stayed with them (LMM of exploration: LRT: χ^2^(1) = 8.282; p = 0.004; H → U β ± SE = 0.728 ± 0.235; t(15) = 3.091; p = 0.007; Fig. 1D) suggesting that the H had a secure base effect, thus in her presence the individuals were calmer. We also found that wolves showed more exploration in general (LMM of exploration: LRT: χ^2^(1) = 10.357; p = 0.002; D → W β ± SE = 1.102 ± 0.309; t(17) = 3.563; p = 0.002) probably due to their more complex environment. Finally, both dogs and wolves interacted more with U when she stayed with them compared to the H (GzLMM of interaction: LRT: χ^2^(1) = 7.549; p = 0.006; H → U β ± SE = − 0.3643 ± 0.132; z(6) = − 2.762; p = 0.006), and also both species interacted less with the staying person in general during the second trial (GzLMM of interaction: LRT: χ^2^(1) = 5.668; p = 0.017; 1st → 2nd β ± SE = − 0.316 ± 0.132; z(6) = − 2.400; p = 0.016).

Escape behaviours were nearly absent in dogs, thus we only tested the effect of the leaving person in the wolf trials in this case. We have found no difference neither in case of the escape scores, nor comparing the phases when the two people stood beside each other, except lip licking showing moderate stress in wolves (see Supplementary Table 4).

## Discussion

Our results shed new light on the domestication of the dog and provide empirical evidence that adult, intensively socialized wolves show similar behaviours than dogs during the separation from their handler at an unfamiliar place and that they are able to form individualized social bonds with humans, including features of attachment such as separation stress, contact seeking and secure base. Although the ontogeny of the behaviour complex in wolves is still not clear it is less likely that this behaviour stems from the mother-infant relationship. Considering that wolf pups at an early age probably do not form such relationships with their mother, nor with their human caregivers—at least at the age of 16 weeks—and the fact that in our study the Handler in most of the cases was not the original caregiver of the subjects, it seems that these bonds are formed only at a later age^1,14^. These results raise the possibility that the attachment towards humans in dogs might have originated from the social bond between the members of the wolf pack^27^, that has a very similar social structure to human families, in which companion dogs live today^19^. Naturally, here the role of extended socialization needs to be emphasized, that eventually helps wolves to form social bond with humans. However, the alternative hypothesis that such a complex behaviour emerged only as a result of the intensive human socialization during the ontogeny of our wolf subjects (i.e. without any trace already in the natural intraspecific social behaviour of the species) is very unlikely^12^.

Though, the main idea behind the SST, that the subjects are exposed to a moderate stress was fulfilled, as we run our test at an unfamiliar place with the presence of an unfamiliar person and the subjects were separated from their handler we should compare the two methods with caution. We admit that our test in its current form is not suitable to describe all of the suggested manifestations of the attachment behaviour complex and that SST is often considered as the “golden standard”, however the measurement of particular features of attachment is also a common method as naturally it manifests itself in several other situations^9,30,32^. Moreover, even the SST is not perfectly applicable to describe the safe haven effect and also in case of dogs its presence was only proved in a follow up study^9^. The first important difference is the restricted state of our subjects by the leash. Because of this, we decided not to measure the possibility of the increased greeting behaviour towards the handler. However, the greeting behaviour of adult wolves towards their original caregiver was investigated in detail by Ujfalussy et al.^30^. They found that wolves show more intensive greeting behaviour when they were reunited with the familiar person. Second, in the original SST, some toys are offered to the subjects and playing with the owner is also considered as indication of the secure base effect^8^. Although, we found that in the presence of their Handler, wolves showed less signs of stress and explored more their vicinity suggesting its presence, we did not measure playing behaviours. However, it was found that adult hand-reared wolves adjust their behaviour less to human playful signals as dogs do^35^ thus it is possible, that because of this species difference, in wolves’ case measuring playing behaviour with humans is not adequate in any way and its absence or presence is not necessarily associated with the stress level of the subject.

The other important difference between the SST and our paradigm is that the subjects were never left alone completely because of safety reasons. Without a perfect separation, one could argue that the subjects showed stress in the absence of the handler only because of the presence of the stranger who held them (i.e., they might have shown stress in the presence of the unfamiliar person only but not when their handler was around). Our results do not support this explanation on multiple grounds. Firstly, we did not find difference in the wolves’ stress behaviour—except lip licking—nor in their attempts to escape comparing the phases when the two people stood beside each other and—depending on the condition—the handler or the unfamiliar person held the leash. Though, as it was expected—based on the increase of the number of lip lickings, what is known as a stress signal in dogs^36^ (and possibly in other canids too)—the proximity of the stranger caused a moderate level of stress in wolves, but possibly not intense enough to overshadow the attachment behaviour. During the separation, the subjects pulled the leash and gazed more towards the hiding place of the handler but not towards any other direction what they would have done in case of escape attempts. The presence of the stranger, therefore, did not seem to cause too intense stress, consequently, the wolves did not try to escape from her. On the other hand, if we assume that the subjects indeed experienced intensive stress because of the presence of the unfamiliar person, this manifested only during the separation from the handler and not when she was still present. This suggests that the handler might have had a calming effect on them which also can be interpreted as a secure base effect. Moreover, if they indeed experienced such intensive stress because of the stranger but still tried to re-establish the contact to seek protection from their handler (i.e.: pulled the leash towards her hiding place) then in this case it might be considered as a sign of safe-haven. Finally, dogs and wolves showed very similar behavioural patterns during the tests. As we can assume that the inner state behind the behavioural reactions is similar in case of two such closely related species^37^, there is a little chance that dogs would react so intensively only to the presence of a stranger. The dog subjects were normally socialised family dogs, thus they are used to meet new people regularly during their everyday life. Consequently, it is very likely that the observable behaviour during the separation were rather caused by the absence of the handler than because of the fear from the stranger.

Although there was no difference in the wolves’ and dogs’ reaction to the separation from the two people, we have found several species-specific differences. Dogs showed more contact seeking behaviour towards the leaving person regardless of her familiarity which can be explained by the fact that they are generally more attracted towards humans than wolves^38^. Meanwhile, they pulled the leash less frequently which is not surprising as usually dogs are trained not to pull the leash but also wolves were found to be generally more persistent than dogs^39,40^. During the test wolves showed more explorative behaviour. Although it is known that wolves explore novel objects longer^29^, we cannot exclude that this difference emerged as a result because of the different environments of the two test sites. Lastly, we found that wolves showed less stress during their second trial, possibly because they became habituated to the test reflecting in lower stress by their second condition. Regarding the contact with the staying person we found that both dogs and wolves spent more time interacting with the unfamiliar person. Although during the SST these interactions are considered as comfort seeking behaviours^8^, in this situation probably it might have been rather caused by the novelty effect of the unfamiliar person^30^. This explanation is supported by the habituation across the two conditions. We have to note the large individual variance in dogs’ stress related behaviours, while other features of attachment seem to be more homogeneous^8,41^ in line with our findings. This, on one hand might be due to the various breed composition of our sample, as it is known that separation behaviour is affected by breed function^42^. Alternatively (but not exclusively) due to several various factors (including individual experiences, genetics, environmental effects)^26^ dogs exhibit highly variable sensitivity to separation and exhibit wide range of stress indicating behaviours^43^, and this is reflected even in our small sample though we did not measure dogs with owner-reported separation related problems.

It is important to mention that our study has its limitations. There are differences in our subjects’ raising and keeping conditions and we tested them at different places. However, we argue that to answer our main question—namely whether wolves are able to form these special relationships with humans—the direct comparison with dogs is not needed. We still decided to measure dogs as an important control group to make sure that our newly developed paradigm indeed suitable to trigger these manifestations of the attachment behaviour-complex. As we found surprisingly little differences in the two species’ behaviour during our test, we argue that despite the differences our interpretation is still well-grounded.

In conclusion, our results suggest that the basis of the ability to form individualized heterospecific bonds during adulthood might have been present in the common ancestor of the wolf and the dog. The attachment behaviour complex is considered to be the core of the dog’s social competence, thus it is reasonable to assume that its’ elements might have been already present before or during the early domestication. Presumably there was a strong selection pressure on the development to form these relationships with humans easily without intensive socialisation and from early puppyhood. Meanwhile dogs were selected to become more and more dependent on humans^8^, the attachment behaviour was re-directed towards humans through genetic changes and dogs became predisposed to form these bonds easily. Consequently, they are able to establish it already during puppyhood^14^. Moreover, while wolves usually do not meet new individuals regularly—thus it is not known how the ability to form these bonds change through their lifetime—dogs are able to form it any point at their life, during adulthood and even after minimal human contact^14,44,45^. Our results suggest that the precursors of dog–human attachment was already present in the common ancestor with wolves providing the basis and organizing the emergence of more complex socio-cognitive skills of the dog and the competence of the formation of successful interspecific social groups with humans^10^.

## Methods

### Subjects

The grey wolves (*Canis lupus*; N = 11) were more than 1.5-year-old, from 6 litters (N = 6 females and N = 5 males; mean age: 6.3 years; range 1.5–15 years; N = 3 intact, N = 9 neutered; for details see Supplementary Table 1). The wolves were individually hand-reared (except for two that was also hand-reared but together with its siblings) from the age of 4–14 days they were bottle-fed, and they spent 22–24 h per day with their caretakers at their homes. At their foster homes, they were intensively socialized and accompanied with their caretakers during their everyday life while they met new places, strangers and social situations regularly. At the age of 3–4 months, the wolves were integrated to live together in packs. While, throughout their adulthood the wolves were still regularly trained and they were used to travel by car and meet new places and people. One wolf due to deviations from the protocol because of Experimenter error and one individual’s second trial due to external disturbance had to be excluded, resulting in 10 wolves included in the analysis.

The Handler (H; young woman: DU) knows all the wolves from puppyhood, she met them on a weekly basis, and was the caregiver of four pups, too. As adults, the wolves live in packs formed from 3 to 6 individuals in large enclosures and they are still in everyday contact with their handler.

The dogs (*Canis familiaris*); N = 9 (7 females and 2 males; mean age 5.1 years; range 1.5–11.5 years; 3 intact, 6 neutered; for details see Supplementary Table 1) were normally socialized, healthy family dogs free from owner-reported separation related behaviour problems, recruited with their owners through social media. In their cases the owner played the role of the Handler during the tests.

The Unfamiliar (U) person was a young woman (VB) who has never met the wolves or the dogs before.

### Ethics declarations

The Animal Welfare Committee of Eötvös Loránd University approved and accepted the experimental protocol (Ref. no.: PEI/001/1058-4/2015) and the tests were performed in accordance with the Hungarian regulations on animal experimentation and the Guidelines for the use of animals in research described by the Association for the Study Animal Behaviour (ASAB).

### Location

We tested the wolves in a forest area that was unfamiliar to them, whereas the dogs were tested outdoors, in a silent parking lot next to the university buildings.

### Set-up

Wolves were accustomed to traveling by car in crates, and on the testing day they were transported to the test site in groups of 2–3 individuals. Before the test, the H put the leash on the focal individual while the others stayed in their crates. The wolves were accustomed to wearing and walking on leash. Dogs arrived to the test site with their owners.

During the test, the subjects wore two leashes for safety reasons. One was shorter (1.5 m) and they could pull it, while the other one was longer (15 m) and it remained loose the whole time. If a wolf would chewed through the shorter leash or the U/H would have decided that it was not safe anymore to hold it back, she could release it, while the longer leash remained still on. When the subject arrived at the test site, two cameras (Sony FDR-AX33) and two shotgun microphones (Sennheiser ME-65 with K6 power module) linked to a Zoom H5 handheld recorder were already set and each device was standing on low tripods. The Experimenter (TF), who handled the sound recording device was already there hiding behind a V-shaped barrier (a wooden panel, width: 160 cm; height: 125 cm), motionless not to attract the attention of the subjects.

### Experimental conditions

- U leaves the subject, H holds the leash
- H leaves the subject, U holds the leash

All subjects were tested in both conditions, half of them started with one and the other half with the other condition. A minimum 5-min break was kept between conditions, while U and H left the test area with the focal animal and took a short walk with them.

### Experimental procedure

Before the separation the H and the U stood beside each other motionless while, depending on the condition, the U or the H held the leashes (Phase 1: Baseline). After 30 s elapsed, the one of them who was assigned to leave said “goodbye” to attract the attention of the subject and walked away (Phase 2: Leaving). After a 50 m long walk along a path coming around a curve she disappeared from the view of the subject (Phase 3: Absence). After 3 min (started when the U/H left and timed by herself with a stopwatch), she came back (Phase 4: Returning) and greeted the subject (Phase 5: Greeting). During the test the person (U or H) who stayed with the subject to help the later synchronization of the audio and video recordings indicated vocally/audibly (with quiet coughing) when the other left, remained out of sight and when reappeared, but otherwise stood silently avoiding interacting with the focal animal.

### Behavioural coding and data analysis

Behavioural coding of video recordings was carried out using Solomon Coder (beta 17.03.22 copyright by András Péter). Supplementary Table 3 shows the coded variables. In another case the leaving person remained visible during the separation thus we analyzed only the first two phases. Due to the limited viewing angle of the cameras some behaviours were not always clearly visible to be coded reliably. Such variables were excluded from the analysis on a case-by-case basis; exclusion criteria were set as the given behaviour being unobserved for more than ten percent of the total time of the phase. The reliability of the coding was checked by re-coding 4 (20%) videos by an independent coder. Kappa statistics were calculated for each behaviour category by taking 20% random samples from the coding timepoints 100 times and calculating the Cohen kappa values for each sampling and averaging them. The overall mean kappa value was 0.763, indicating reliable coding.

To characterize the behaviour of the subjects in the absence of the disappearing person we calculated five Separation scores by summing the percent time of the selected behaviours then averaging these over the Leaving and Absence phases (i.e., these two phases were treated as one in the analyses). The scores were (1) stress, containing whining, panting, other vocalizations and movement, (2) contact seeking, containing orientation at, and leash tension towards the Disappearing person, (3) exploration, containing sniffing and object exploration, and (4) escape scale, containing general leash tension and chewing. Additionally, (5) interaction scale was calculated by summing the frequencies of orientation at and physical contact with the Staying person. Finally, we analyzed frequencies of mouth licking and pulling the leash separately. These calculated scores and frequencies were used as response variables in further analyses. While in wolves barking is a rare vocalization type and typically they do not bark in isolation^46^ in case of dogs it appears in the absence of the owner in a similar outdoor situation^32,47^. However, in our sample only two dogs barked during the test thus we decided not to include barking in our analysis at all.

All analyses were performed in R statistical environment (4.0.0^48^) using RStudio (1.2.5042^49^). Scores were normalized by boxcox transformation when deviance from normal distribution was detected. We applied linear mixed-effects models LMMs (nlme^50^) on each score adding the individuals (subject ID) as random factor and including Trial (1st or 2nd), Leaving Person (U or H) and species as main factors and their two- and three-way interactions. Then we applied AIC-based backwards elimination to find the parsimonious model. Frequencies of certain point-like behaviours (mouth licking, pulling the leash) were analyzed with GzLMM (lme4^51^) with Poisson distribution and log link. Otherwise, the initial model structures and the selection process were the same as above. Tukey test was used for post-hoc comparisons in case of interaction effects (emmeans^52^).

## Supplementary information

Supplementary Dataset.

Supplementary Information.

## Data Availability

All raw and derived data are available as Supplementary material.
