# Supplementary material for: Adult, intensively socialized wolves show features of attachment behaviour to their handler
Source: Sci Rep. 2020 Oct 14;10:17296. doi: 10.1038/s41598-020-74325-0 (PMC7560749; doi:10.1038/s41598-020-74325-0)
Supplement: Supplementary file 2 — Supplementary Information. [file 41598_2020_74325_MOESM2_ESM.pdf]

Adult, intensively socialized wolves show features of attachment behaviour to their handler

Rita Lenkei[1], Dóra Újváry[1], Viktória Bakos[1] and Tamás Faragó[1*]

[1]*Department of Ethology, Eötvös Loránd University, Budapest*

* Correspondence to: mustela.nivalis@gmail.com

Supplementary Materials:

Supplementary Figure 1.

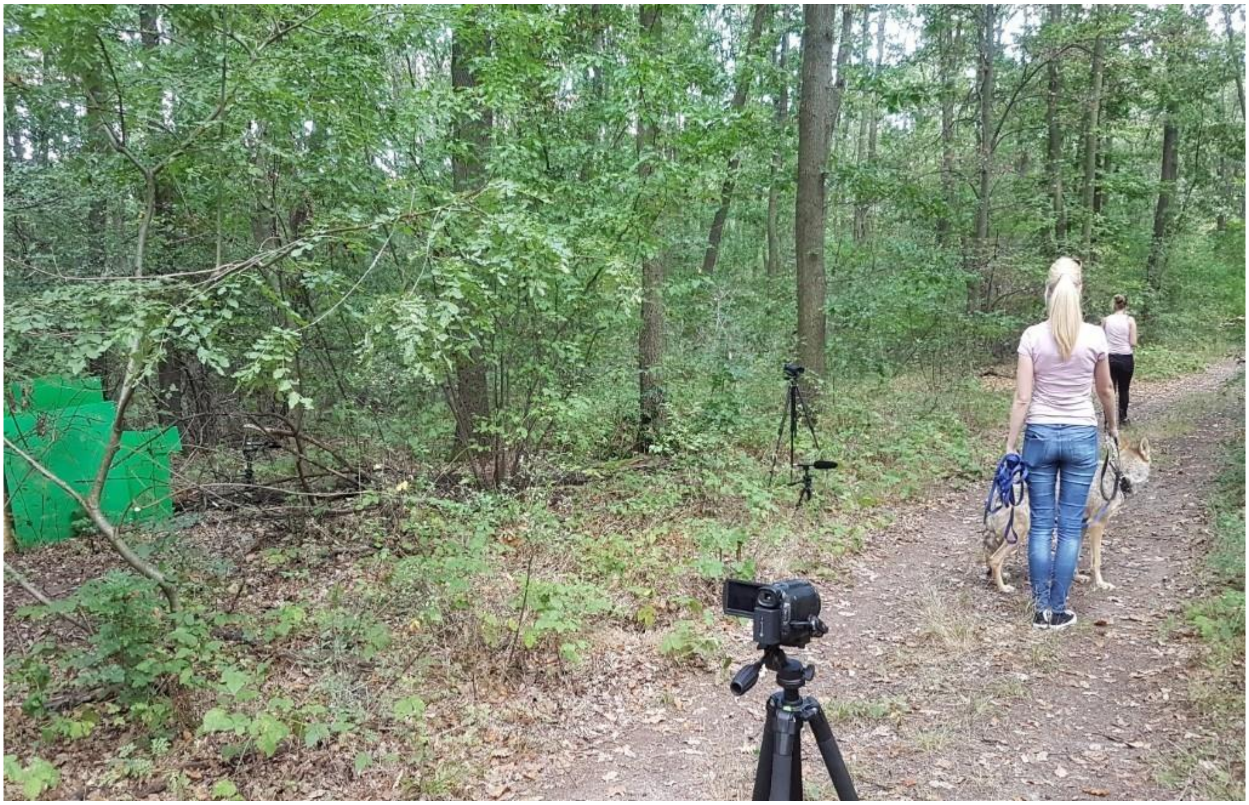

The test setup for wolves

Supplementary Table 1.

Name	Litter ID	Sex	Reproductive status	Caregiver	Rearing	Age (years)	Species/Breed
**Babóca**	1	male	neutered	Other	Hand-reared individually	14	wolf
**Dakota**	2	male	intact	UD	Hand-reared individually	14	wolf
**Hella**	3	female	neutered	Other	Hand-reared individually	1,5	wolf
**Hogan**	4	male	neutered	Other	Hand-reared with siblings	9,5	wolf
**Hupa**	3	female	neutered	Other	Hand-reared individually	1,5	wolf
**Nelson**	5	male	neutered	UD	Hand-reared individually	9,5	wolf (excluded)
**Runa**	3	female	neutered	Other	Hand-reared individually	1,5	wolf
**Zabu**	3	male	neutered	UD	Hand-reared individually	1,5	wolf
**Zira**	3	female	neutered	Other	Hand-reared individually	1,5	wolf
**Zorba**	6	male	intact	UD	Hand-reared with siblings	15	wolf
**Zordon**	3	male	neutered	Other	Hand-reared individually	1,5	wolf
**Bella**	-	female	intact	-	-	1,5	Irish Setter
**Cheyenne**	-	female	neutered	-	-	11,5	Belgian Shepherd
**Csoki**	-	male	neutered	-	-	4,5	Mixed
**Jafar**	-	male	intact	-	-	3	White Swiss Shepherd Dog
**Kida**	-	female	neutered	-	-	5,5	Boxer
**Leila**	-	female	neutered	-	-	3	Golden Retriever
**Lenke**	-	female	intact	-	-	5,5	Bichon Havanese
**Luca**	-	female	neutered	-	-	9	Mixed
**Molly**	-	female	neutered	-	-	2,5	Wirehaired Dachshund

Basic information of the subjects

Supplementary Table 2.

Phase	Name	Time	Description	Start - End
Phase 1	Baseline	30 s	The H and the U stood beside each other without interacting with the subjects meanwhile the one who stayed with the subject held the leash.	The H/U set the stopwatch – After the 30 sec. elapsed
Phase 2	Leaving	approx. 40 s	The H/U said goodbye to the subject and walked away disappearing from the sight.	The last physical contact with the subject after saying good-bye – When the hiding person disappeared (indicated by the staying person)
Phase 3	Absence	approx. 140 s	The H/U was out of sight.	When the hiding person disappeared (indicated by the staying person) –When the hiding person reappeared (indicated by the staying person)
Phase 4	Returning	approx. 40 s	After the 3 minutes elapsed she reappeared and walked back.	When the hiding person reappeared (indicated by the staying person) - The first physical contact with the subject (if the subject showed avoidance then the moment when the H/U were the closest to him/her)
Phase 5	Greeting	10 s	The H/U greeted and petted the subject.	The first physical contact with the subject (if the subject showed avoidance then the moment when the H/U were the closest to him/her) – After the 10 sec. elapsed

The description of the test phases. In the analysis Leaving and Alone phases were merged to form Separation phase.

Supplementary Table 3.

Category	Name	Type	Definition
Position	Move	Duration	The wolf/dog moved (at least two legs was moved)
	Stand	Duration	The wolf/dog was standing (4 paws were on the ground)
	Lie	Duration	The wolf/dog was lying
	Sit	Duration	The wolf/dog was sitting
Vocalisation	Whine	Duration	The wolf/dog made a high-pitched vocalization
	Pant	Duration	A repeated noise made by the wolf/dog, which sounds like loud and fast breath taking
	Growl	Duration	The wolf/dog made a low-pitched noisy vocalization
	Other	Duration	The wolf/dog made other types of vocalizations which were not in the other categories
Object exploration		Duration	The wolf/dog was sniffing the ground or any other object around
Sniffing		Duration	The wolf/dog was sniffing the air (head held up, movement of the nose was visible)
Orientation	Staying person	Duration	The wolf's/dog's head was turned towards the staying person, excluding sniffing and exploration
	Leaving person	Duration	The wolf's/dog's head was turned towards the leaving person or towards the way where she left, excluding sniffing and exploration
Physical contact contact	Staying person	Duration	The wolf was in physical contact with the U (only coded if the wolf initiated it)
	Leaving person	Duration	The wolf was in physical contact with the H (only coded if the wolf initiated it)
Leash pull	Towards	Frequency	The wolf/dog pulled the leash once towards the leaving person
	Other	Frequency	The wolf/dog pulled the leash once towards any other direction
Leash tension	Towards	Duration	The leash was tense towards the leaving person, excluding sniffing and exploration
	Other	Duration	The leash was tense towards any other direction, excluding sniffing and exploration
Leash chew		Duration	The wolf/dog chewed the leash (it was in his/her mouth)
Mouth lick		Frequency	The wolf/dog licked his/her mouth (the tongue was visible)

The coded behaviour responses

Supplementary Table 4.

Behaviour		B	SD	df	t/z	p
Stress	leaving p	-0.4102734	0.4144234	17	-0.989986	0.3361
	species	0.4195540	0.5520456	17	0.759999	0.4577
	leaving p:species	-0.1137507	0.5712432	17	-0.199128	0.8445
Exploration	leaving p	-0.992519	0.8603247	16	-1.153656	0.2656
	species	0.288162	0.9741752	17	0.295801	0.7710
	leaving p:species	1.614836	1.2053617	16	1.339710	0.1991
Escape	leaving p	-0.3246345	0.5013925	9	-0.647466	0.5335
Interaction	leaving p	0.04652	0.30508		0.152	0.879
	species	-0.20673	0.55791		-0.371	0.711
	leaving p:species	0.01410	0.46303		0.030	0.976
Lip lick fr.	leaving p	0.2877	0.7638		0.377	0.7064
	species	1.9095	0.6236		3.062	0.0022
	leaving p:species	-1.0986	0.8660		-1.269	0.2046

Comparison of the baseline phases between the trials

Supplementary Data (separate file)

All raw behaviour data of the dogs and wolves and the calculated scores used in the analysis.
